# Supplementary material for: Mitochondrial stress disassembles nuclear architecture through proteolytic activation of PKCδ and Lamin B1 phosphorylation in neuronal cells: implications for pathogenesis of age-related neurodegenerative diseases
Source: Front Cell Neurosci. 2025 Apr 17;19:1549265. doi: 10.3389/fncel.2025.1549265 (PMC12043892; doi:10.3389/fncel.2025.1549265)
Supplement: Supplementary file 1 [file Data_Sheet_1.docx]

Supplementary materials

Mitochondrial Stress Disassembles Nuclear Architecture through Proteolytic Activation of PKCδ and Lamin B1 Phosphorylation in Neuronal Cells: Implications for Pathogenesis of Age-related Neurodegenerative Diseases

**Adhithiya Charli^1^; Yuan-Teng Chang^2^; Jie Luo^1^; Bharathi Palanisamy^1^; Emir Malovic^1^; Zainab Riaz^2^; Cameron Miller^2^, Manikandan Samidurai^2^; Gary Zenitsky^2^; Huajun Jin^2^; Vellareddy Anantharam^2^; Arthi Kanthasamy^2^; Anumantha G Kanthasamy^1,2*^**

^1^Parkinson’s Disorder Research Laboratory, Iowa Center for Advanced Neurotoxicology, Department of Biomedical Sciences, Iowa State University, Ames, IA 50011

^2^Department of Physiology and Pharmacology, Isakson Center for Neurological Disease Research, University of Georgia, Athens, GA 30602

***Correspondence:** Anumantha Kanthasamy, Professor, Johnny Isakson Chair, Georgia Research Alliance Eminent Scholar, and Director, Center for Neurological Disease Research, Department of Physiology and Pharmacology, 325 Riverbend Road, Center for Molecular Medicine Bldg, University of Georgia, Athens, GA 30602. Telephone: (706) 542-2380; Fax: (706) 542-4412; Email: anumantha.kanthasamy@uga.edu


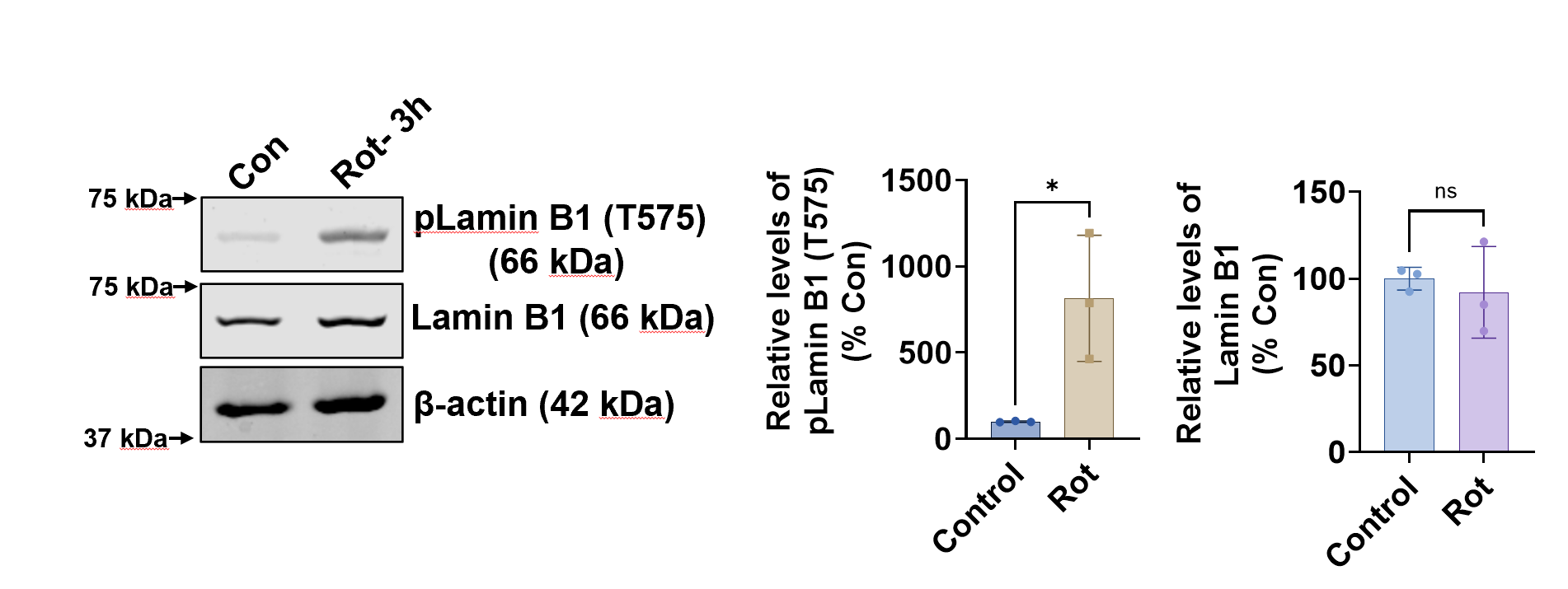


**Supplemental Figure 1:** Mitochondrial inhibition triggers Lamin B1 phosphorylation in the DAergic neuronal cell culture model. N27 cells were treated with rotenone (Rot, 1 µM) for 3 h. Representative immunoblots of phospho-Lamin B1 (T575) showing phosphorylation of Lamin B1 at T575 induced by rotenone. β-Actin was used as a loading control. Data shown represent mean ± SD performed in triplicate (*p≤0.05; ns, not significant). n=3.


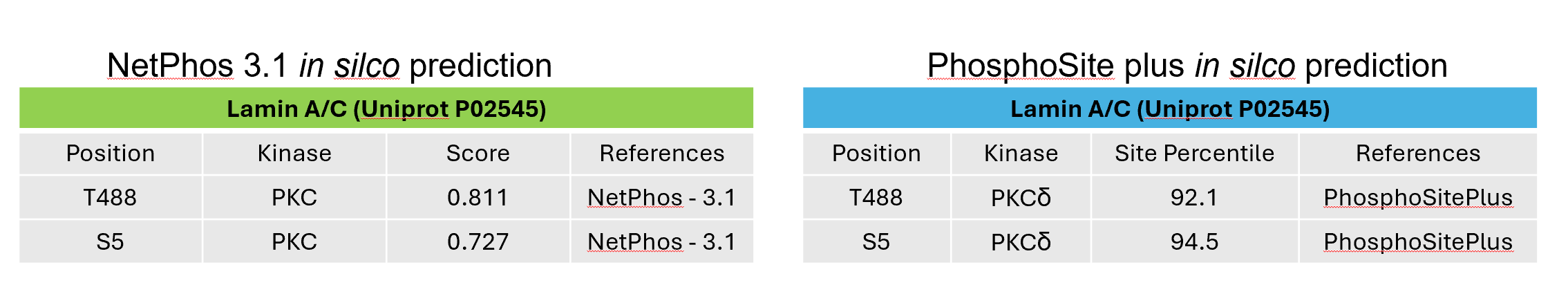


**Supplemental Figure 2:** Prediction of Lamin A/C phosphorylation sites. *In silico* phospho site-matching analyses using the online tools NetPhos Server 3.1 (left) and PhosphoSitePlus (right) showing the potential PKCδ phosphorylation sites on Lamin A/C.
